# Supplementary material for: The Short- and Long-Range RNA-RNA Interactome of SARS-CoV-2
Source: Mol Cell. 2020 Dec 17;80(6):1067–1077.e5. doi: 10.1016/j.molcel.2020.11.004 (PMC7643667; doi:10.1016/j.molcel.2020.11.004)
Supplement: Document S1. Figures S1–S5 and Table S1 [file mmc1.pdf]

Molecular Cell, Volume *80*

Supplemental Information

The Short- and Long-Range RNA-RNA Interactome of SARS-CoV-2

Omer Ziv, Jonathan Price, Lyudmila Shalamova, Tsveta Kamenova, Ian Goodfellow, Friedemann Weber, and Eric A. Miska

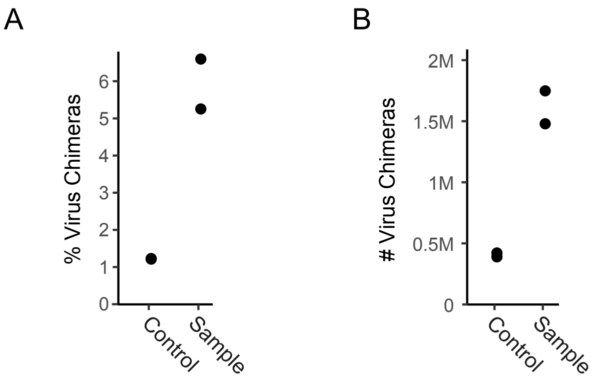

Supplementary Figure 1, related to Figure 1. The COMRADES method.
(A) Percentage of chimeric reads in the samples and control experiments.
(B) Number of chimeric reads in the samples and control experiments.

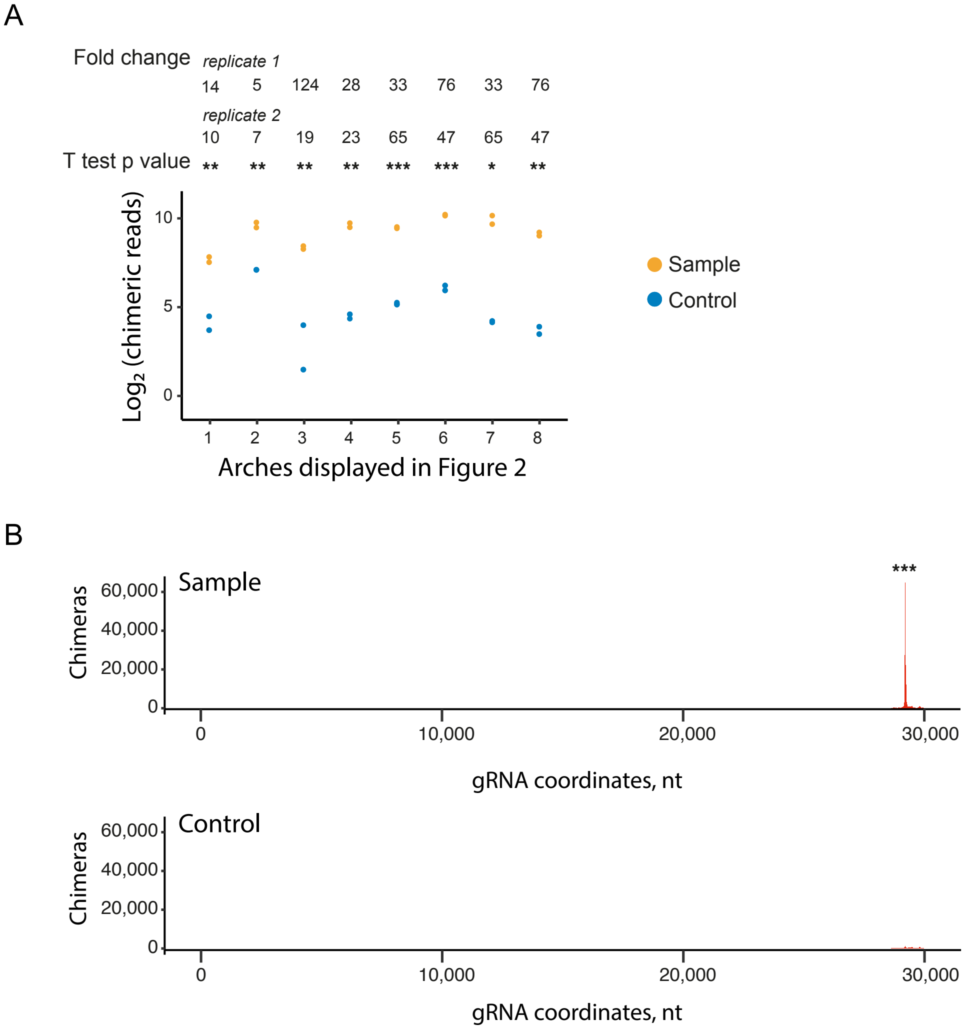

Supplementary Figure 2, related to Figure 3. Long-range RNA-RNA interactions along the SARS-CoV-2 gRNA and N ORF sgmRNA
(A) Number of chimeric reads supporting the arches shown in Figure 2 in COMRADES samples and controls libraries. Log_2 values are shown. Fold changes represent the ratios of chimeras in sample / chimeras in control for each biological replicate. T test p values indicate the significant of each arch (* < 0.1, ** < 0.05, *** < 0.01).
(B) Interactions of the leader sequence with a downstream position in ORF N sgmRNA. Number of chimeric reads supporting this base-pairing is shown. *** denotes T test p value < 0.01

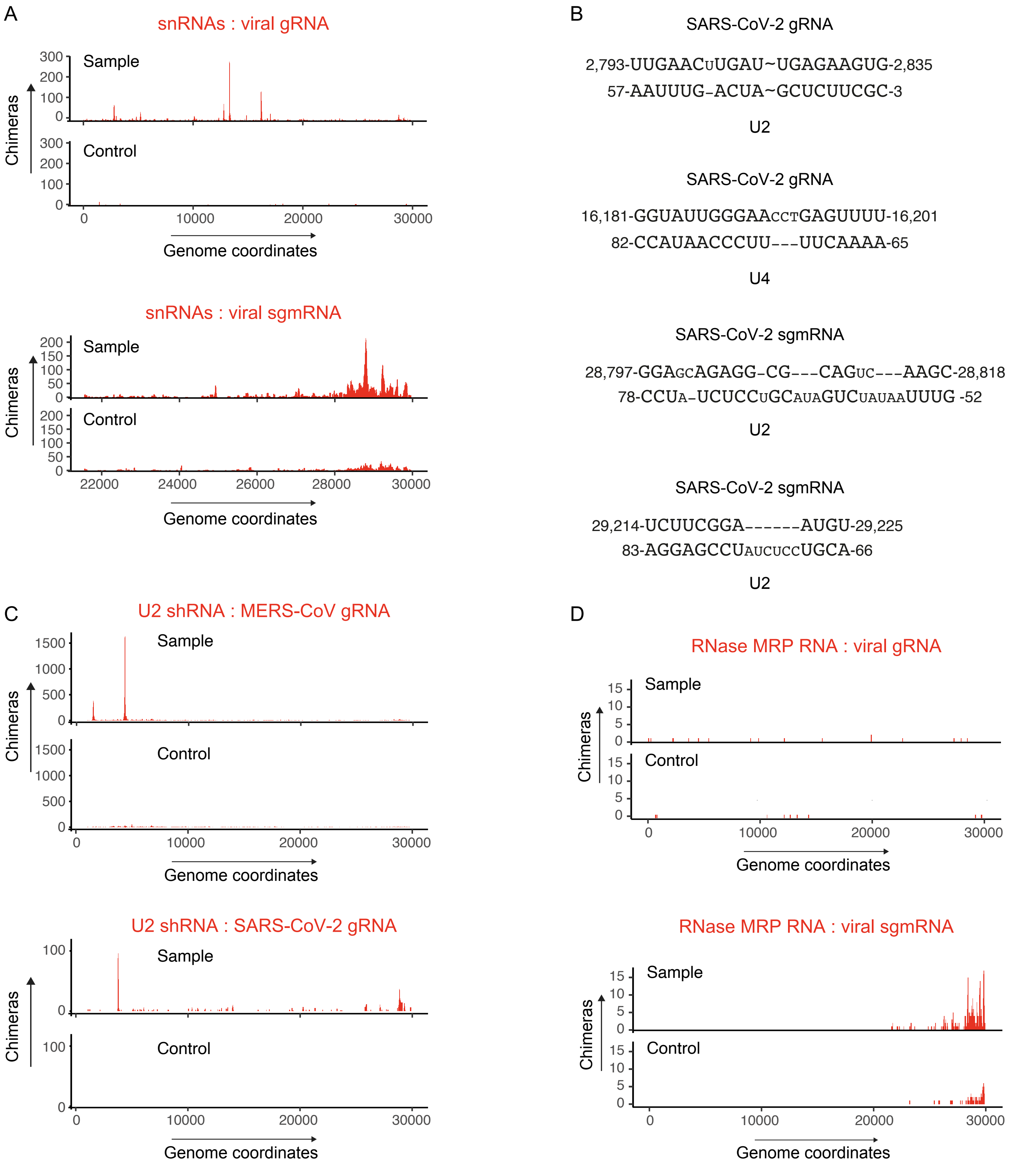

Supplementary Figure 3, related to Figure 4. Interactions between cellular and viral RNA
(A) snRNAs binding positions along the SARS-CoV-2 gRNA (top) and sgmRNA (bottom) and their COMRADES-controls.
(B) Base-pairing models for the interactions between viral RNA (top strand) and host snRNAs (bottom strand)
(C) U2 snRNA binding position along MERS-CoV gRNA (top) and SARS-CoV-2 gRNA (bottom) and their COMRADES controls
(D) RNase MRP binding positions along the SARS-CoV-2 gRNA (top) and sgmRNA (bottom) and their COMRADES controls.

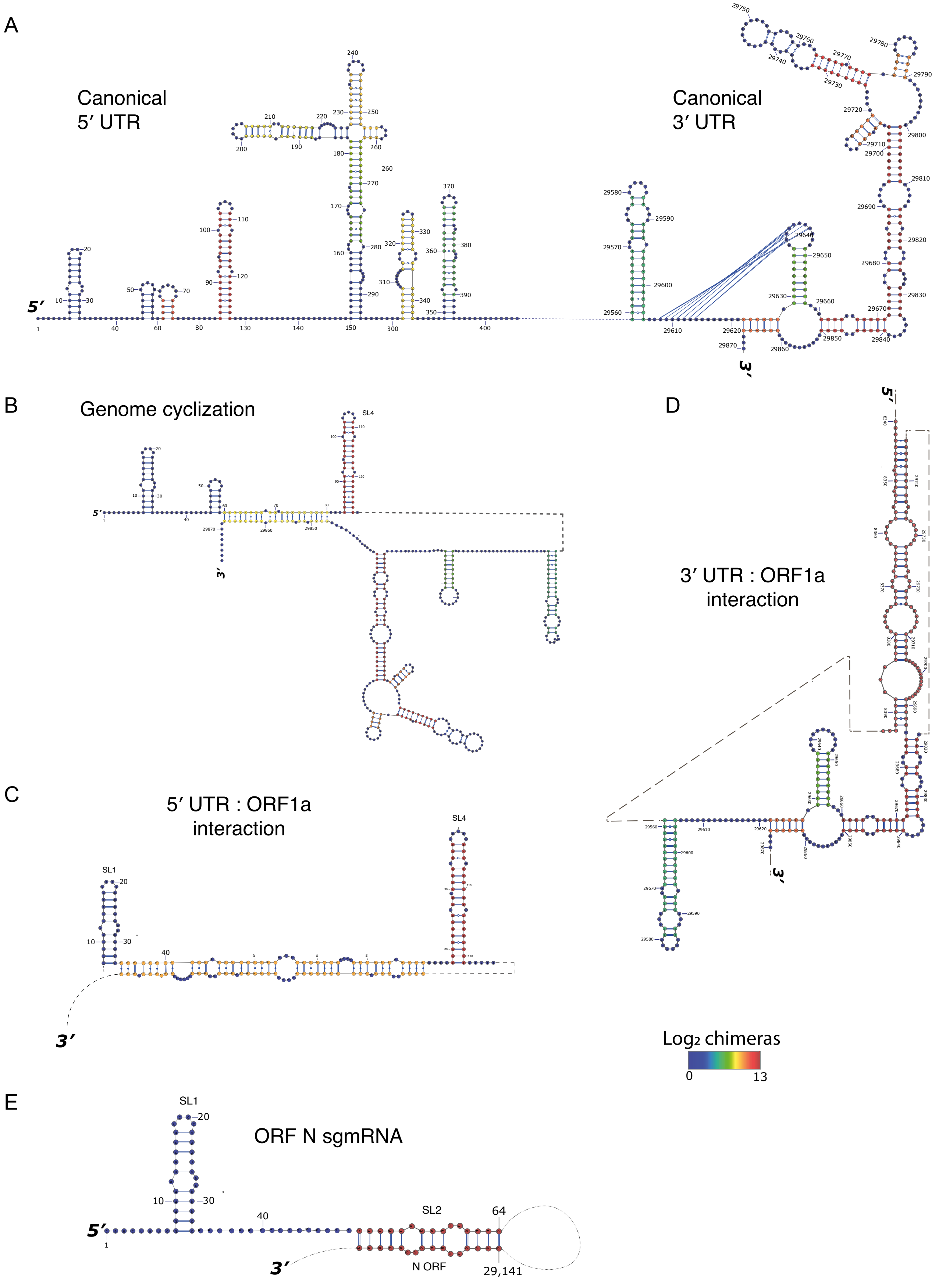

Supplementary Figure 4, related to Figure 5. The UTRs of SARS-CoV-2 adopt alternative conformations inside cells

(A-E) Detailed representation of the canonical UTRs structure (A); genome cyclization (B); UTRs binding to ORF1a (C,D); and ORF N sgmRNA conformation (E). Colour code represents the number of non-redundant chimeric reads supporting each base-pair.

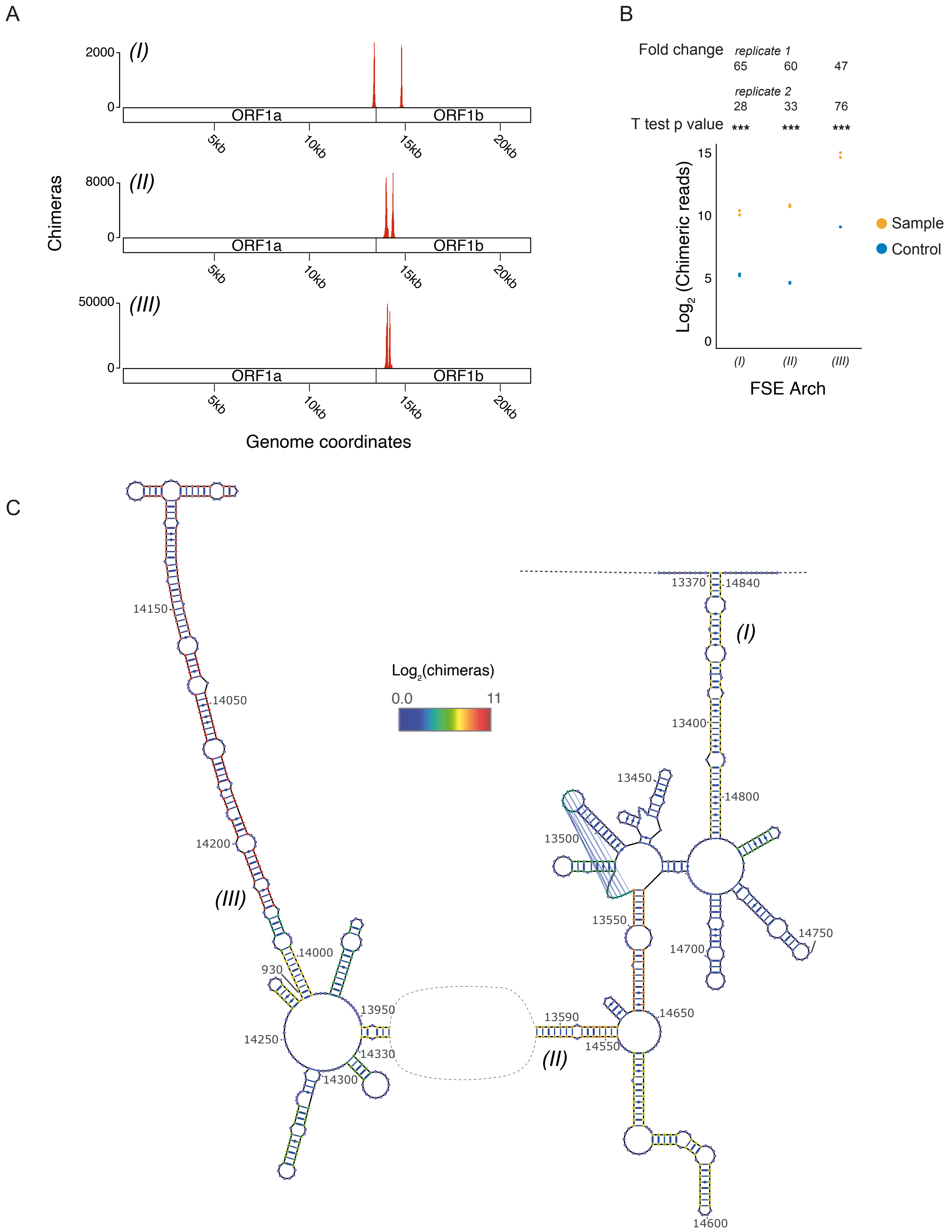

Supplementary Figure 5, related to Figure 6. The structure of the SARS-CoV-2 ribosomal frameshifting element arch (FSE-arch) inside cells

(A) Representation of the left- and right-side of the chimeric-reads supporting the FSE-arch. *(I)*-*(III)* correspond to the gRNA positions marked in (C).

(B) Number of chimeric reads supporting the FSE-arch in samples and controls. Log_2 values are shown. Fold changes represent the ratios of chimeras in sample / chimeras in control for each biological replicate. T test p values indicate the significant of each position (*** < 0.01). *(I)*-*(III)* correspond to the gRNA positions marked in (C).

(C) Detailed representation of the FSE-arch structure. Colour code represents the number of non-redundant chimeric reads supporting each base-pair.

Supplementary Table 1. Prevalence of chimeras supporting the long-range interactions shown in Figure 3.

Arch name	Long-range chimeras[a]	Short-range chimeras[b]	Ratio of Long-range / Short-range chimeras
1	442	14,506	3.0%
2	1,873	5,980	31.3%
3	704	5,980	11.8%
4	1,690	13,546	12.5%
5	1,529	9,050	16.9%
6	2,493	7,447	33.5%
7	2,102	16,531	12.7%
8	1,193	11,766	10.1%

[a]Long-range chimeras supporting each arch
[b]Chimeras supporting alternative, short-range interactions within the arch regions
